# Using the behaviour change wheel and person-based approach to develop a digital self-management intervention for patients with adrenal insufficiency: the *Support AI* study protocol

**DOI:** 10.3389/fendo.2023.1207715

**Published:** 2023-06-30

**Authors:** Sofia Llahana, Kathleen Mulligan, Shashivadan P. Hirani, Stephanie Wilson, Stephanie E. Baldeweg, Ashley Grossman, Christine Norton, Philippa Sharman, Pat McBride, Stanton Newman

**Affiliations:** ^1^ School of Health & Psychological Sciences, City, University of London, London, United Kingdom; ^2^ Department of Diabetes and Endocrinology, University College London Hospitals National Health Service (NHS) Foundation Trust, London, United Kingdom; ^3^ Community Health Newham, East London National Health Service (NHS) Foundation Trust, London, United Kingdom; ^4^ Centre for Human Computer Interaction (HCI) Design, School of Science and Technology, City, University of London, London, United Kingdom; ^5^ Centre for Obesity and Metabolism, Department of Experimental and Translational Medicine, Division of Medicine, University College London, London, United Kingdom; ^6^ Neuroendocrine Tumour (NET) Unit, Royal Free Hospital, London, United Kingdom; ^7^ Green Templeton College, University of Oxford, Oxford, United Kingdom; ^8^ Florence Nightingale Faculty of Nursing, Midwifery and Palliative Care, King’s College London, London, United Kingdom; ^9^ The Addison’s Disease Self-Help Group Patient Advocacy Group, Bristol, United Kingdom; ^10^ The Pituitary Foundation Patient Advocacy Group, Bristol, United Kingdom

**Keywords:** adrenal insufficiency, adrenal crisis, self-management, digital behaviour change intervention, behaviour change wheel, theoretical domains framework, person-based approach

## Abstract

**Introduction:**

Most patients with Adrenal insufficiency (AI) require lifelong glucocorticoid replacement. They need to increase glucocorticoids during physical illness or major stressful situations and require parenteral hydrocortisone in the event of an adrenal crisis. Patients with AI have impaired quality of life and high mortality; approximately 1 in 6-12 patients are hospitalised at least once/year from a potentially preventable adrenal crisis. Adoption of self-management behaviours are crucial; these include adherence to medication, following “sick day rules” and associated behaviours that aid prevention and treatment of adrenal crisis such as symptom monitoring, having extra tablets, carrying a medical-alert ID and injection kit, and self-injecting when necessary. Current patient education is ineffective at supporting self-management behaviour change or reducing adrenal crisis-related hospitalisations. This research study aims to gain an in-depth understanding of the barriers and enablers to self-management for patients with AI and to develop an evidence-based digital self-management behaviour change intervention.

**Methods:**

The study is conducted in accordance with the MRC Framework for developing complex interventions. Underpinned by the Behaviour Change Wheel (BCW), the Theoretical Domains Framework (TDF), and the Person-Based Approach, this research will be conducted in two phases: Phase 1 will involve a sequential qualitative/quantitative mixed-methods study involving focus group interviews followed by a cross-sectional survey with patients with AI recruited from patient advocacy groups and endocrine clinics in the UK. Phase 2 will develop the *Support AI*, a website-based digital behaviour change intervention (DBCI) informed by Phase 1 findings to support self-management for patients with AI. The most appropriate behaviour change techniques (BCTs) will be selected utilising a nominal group technique with an Expert Panel of 10-15 key stakeholders. The design of the *Support AI* website will be guided by the Person-Based Approach using an Agile iterative “think-aloud” technique with 12-15 participants over 3 usability testing iterations.

**Conclusion:**

A theory- and evidence-based digital behaviour change intervention will be developed which will be tested in a feasibility randomised trial following completion of this study. The projected benefit includes cost-effective health care service (reduced hospitalisations and demand for specialist services) and improved health outcomes and quality of life for patients with AI.

## 1 Introduction: self-management for patients with adrenal insufficiency

Adrenal insufficiency (AI) is caused by lack or insufficient production of cortisol from the adrenal cortex. Depending on its cause, AI can be “primary” due to adrenal gland failure (most commonly Addison’s disease), “secondary” due to conditions affecting the hypothalamo-pituitary axis which fails to stimulate cortisol production, or “tertiary” caused by hypothalamo-pituitary-adrenal axis suppression from chronic treatment with corticosteroids (1, 2). Tertiary AI is usually transient and in more than 75% of patients adrenal function recovers within 6 months of stopping corticosteroids, although it can be life-long (3). Most patients with AI require life-long glucocorticoid replacement therapy, commonly hydrocortisone tablets 2-3 times/day. They need to increase glucocorticoids during illness or major stressful situations and require parenteral hydrocortisone in the event of an adrenal crisis to prevent hospitalisation and death; these are called “sick day rules”. Adrenal crisis is a life-threatening acute complication, mainly precipitated by infections, vomiting, diarrhoea, trauma or surgery; patients typically present with profoundly impaired well-being and hypotension, and are often unable to self-inject and may require help from a relative/carer/friend or health professional (4–7).

The standard mortality rate in patients with AI is twofold compared to the general population (8–10), while 1 in 200 patients die from a potentially preventable adrenal crisis (10–12). As a percentage of total diagnosis, deaths from adrenal crisis in the UK are ten times higher than deaths from insulin-dependent diabetes-related ketoacidosis (13). Between one in 6-12 patients with AI are hospitalised at least once a year following an adrenal crisis episode (11, 14, 15). AI poses a significant health burden on patients, their families, and the healthcare system. A UK study in 2013 estimated an annual cost of illness associated with AI at £39.7 million for approximately 20,000 patients (16). Health care cost for patients with AI is four times higher than for the general population (17). More than 60% of patients with AI have impaired quality of life, 40% take sick leave at least quarterly, and in some countries approximately 25% of patients receive a disability living allowance (18–21). The impact from AI and glucocorticoid treatment sequelae can be minimised significantly, and up to 50% of adrenal crisis-related hospitalisations may be prevented with effective self-management (4). This includes treatment optimisation and improved adherence to daily glucocorticoid replacement therapy (alongside mineralocorticoid replacement therapy for “primary” AI and pituitary replacement therapy for “secondary” AI), appropriate glucocorticoid dose adjustment for “sick days”, and timely administration of parenteral hydrocortisone in an adrenal crisis. It also includes adoption of behaviours that aid the prevention or treatment of adrenal crisis, e.g. symptom monitoring, having an extra supply of tablets, wearing a medical-alert ID, carrying a steroid emergency card and an emergency injection kit, and self-injecting when necessary.

These self-management behaviours place a significant burden on patients with AI. Forss et al. reported that 38% of patients with AI found the multiple daily dosing problematic and consequently missed doses (21). Chapman et al. found that only 15% of patients took all their doses as prescribed, 25% of patients took higher doses than advised, while 1 in 25 patients reported prolonged treatment interruptions (22). Non-adherence or interruptions to daily glucocorticoids were found to trigger an adrenal crisis for approximately 5% of patients with AI (14, 23). Glucocorticoid over-replacement exposes patients to medication side effects (21, 22), cardiovascular complications (24), osteoporosis (25), depression and impaired quality of life (20, 21, 26).

Several studies found that 26% – 38% of patients with AI did not adjust their glucocorticoid dose for “sick days” (22, 27–31) and only 60% of patients carried a medical-alert ID (28, 29). Approximately 70% of patients had an emergency hydrocortisone injection kit (5, 15) but only 12% managed to self-inject when they experienced an adrenal crisis (15), while only 19% were trained to self-inject (5). Self-injection reduces the risk of hospitalisations when administered in a timely manner from onset of adrenal-crisis symptoms; 38% of patients who self-injected required hospitalisation versus 73% who were injected by a medical professional due to the delay in waiting for them to arrive (p=0.008) (30).

A recent systematic review of seven patient education studies reported an improvement in patients’ knowledge and self-confidence of managing their AI; this was evaluated using patient self-reported diary-based or cross-sectional questionnaire measures. Although the authors of this systematic review claim that their aim was to evaluate behavioural interventions aiming to prevent adrenal crisis, the included studies were not designed as interventions to bring about behaviour change, i.e. to prevent adrenal crisis, and none was a randomised controlled study. All studies were education-based, focusing on increasing patients’ knowledge about their condition, with the implicit assumption being that information provision will lead to behaviour change. However, assessment of outcomes such as behaviour change, and frequency of adrenal crisis or hospitalisations was not reported. The authors concluded that there is a need to develop behaviour change interventions to support patients with AI to prevent adrenal crisis (32).

Published studies involving patient with AI are limited in that they lack theoretical underpinning for complex intervention development (33). Therefore, there is a need to develop and test an intervention that adopts a broader self-management approach that goes beyond information provision and is informed by theoretical, evidence-based frameworks. Intervention development should involve a dynamic iterative process with stakeholder input throughout development, and should go beyond a narrow focus of increased effectiveness, paying attention to future implementation in the real world (34, 35).

Annual participation in education programmes has been recommended for patients with AI (1, 4, 36, 37) as only 40% retain all the information in these sessions (38). Uptake of education programmes has not been high, with less than 60% taking up the offer of an education programme. This is mainly due to geographical/time constraints and their “one-size-fits-all” approach to organisation of sessions and their content (37, 39, 40).

Face-to-face programmes require extensive specialist time and resources, e.g. one-hour sessions for individual education (40) and 2-3 hours for small patient group education (30, 37, 39), presenting a financial burden for a health service. Digital behaviour change interventions (DBCI) are increasingly recognised for their value and cost effectiveness in supporting self-management in patients with chronic conditions (41–43). There is currently no published evidence of DBCIs in patients with AI. The present study will develop a DBCI, which in line with the goals set in the United Kingdom “more efficient use of specialist services Five Year Forward View” (44) will harness the potential of technology to enable patients with AI to take an active role in their self-management and to enable more efficient use of specialist services. Delivery of the DBCI through a personalised responsive mobile-optimised website can provide an easily accessible and cost-effective platform to complement usual care for all patients with AI, and in this way reduce inequalities in the treatment they receive.

### 1.1 Study aim and objectives

This research study aims to develop a theory-informed digital behaviour change intervention (DBCI) to support self-management in patients with AI.

Objectives:

1. Understand the manner and extent to which patients with AI are engaging with the target self-management behaviours.
2. Identify barriers and enablers and measure their impact on self-management behaviours.
3. Explore how and which of these target behaviours can be addressed through a DBCI.
4. Design a theory-informed DBCI that is acceptable to patients and other key stakeholders.

## 2 Methods and analysis

### 2.1 Theoretical frameworks underpinning the study

This research study is conducted in line with the *Medical Research Council (MRC) Framework for Complex Interventions* which recommends using a systematic and transparent process that involves the following key elements: Development, Feasibility and Piloting, Evaluation, and Implementation (33). In this protocol we discuss the *Development* element. This initially involves a theoretical understanding of the likely process of behaviour change by drawing on existing evidence and analysis via theoretical frameworks through which the intervention could be developed and modelled. Modelling of the intervention involves deciding what should be targeted (determinants of behaviour) and how this can be achieved.

**Figure 1:**
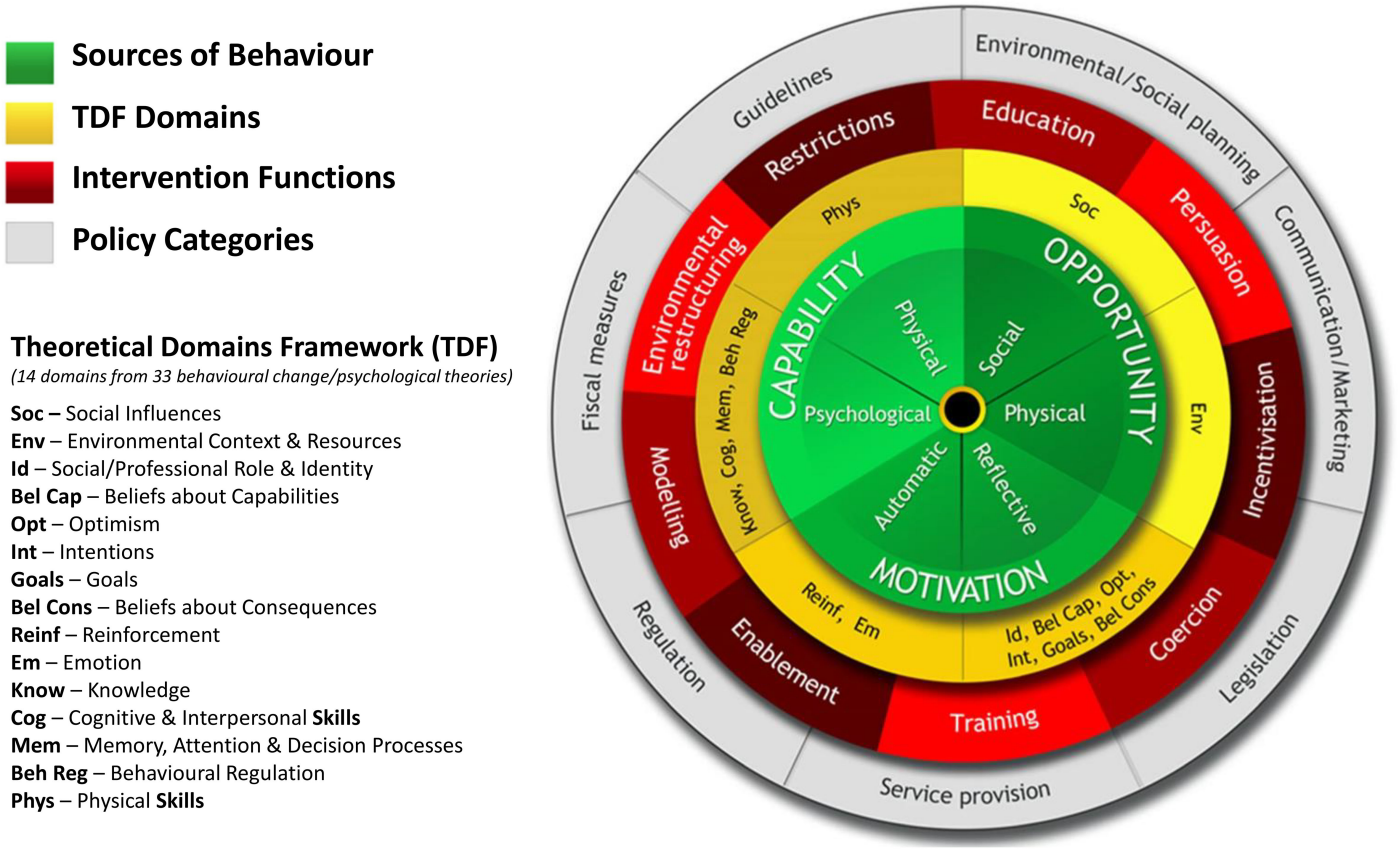
The behaviour change wheel and theoretical domains framework. Reproduced from Michie S, Atkins L, West R (2014) The behaviour change wheel: a guide to designing interventions. Silverback Publishing and Ojo SO et al. (2019) BMC Public Health, 19, 1126, doi.org/10.1186/s12889-019-7468-8.

**Figure 2:**
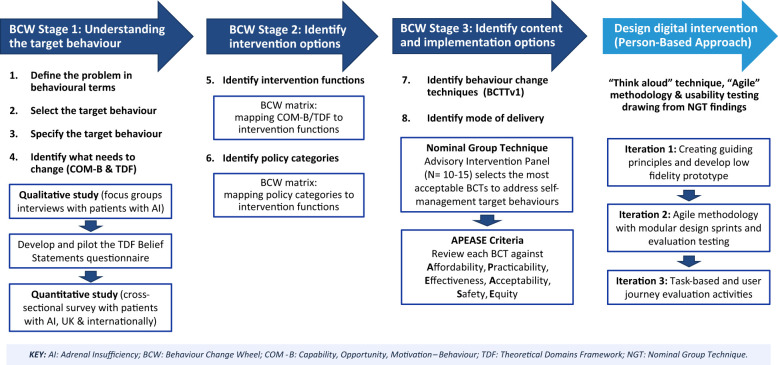
The *Support AI* digital behaviour change intervention development process guided by the Behaviour Change Wheel (BCW) and Person-Based Approach. Reproduced from Michie S, Atkins L, West R (2014) The behaviour change wheel: a guide to designing interventions. Silverback Publishing.

Interventions targeted at changing behaviour need to be informed by theoretical, evidence-based frameworks. We will adopt the Behaviour Change Wheel (BCW) framework (**Figure 1**), developed from 19 existing behaviour change frameworks (45, 46), to underpin the intervention development in 3 stages depicted in **Figure 2**:

•Stage 1: Understand the behaviour,
•Stage 2: Identify intervention options,
•Stage 3: Identify content and implementation options

At the core of the BCW is the COM-B general model of behaviour which explains how behaviours come about at any particular moment based on an individual’s Capability (C) and Motivation (M) and the situations that provide them with the Opportunity (O) to enact or change *Behaviour* (*B*) outlining thus all potential influences on the targeted behaviour (45). The BCW recognises that behaviour change occurs as a result of an interacting system of Intervention Functions, i.e. broad categories of activities aimed at changing behaviour, such as education, persuasion, and Policy Categories, which describe actions on the part of responsible authorities that enable or support the intervention delivery, e.g. service provision, guidelines (45) (**Figure 1**).

Associated with the BCW and COM-B is also the Theoretical Domains Framework (TDF) comprising of 14 domains, each correlating to a COM-B component, drawn from 33 behaviour change and psychological theories (46, 47). The TDF provides a more granular and deeper exploration of the barriers and enablers to behaviour change. The BCW provides guidance on linking the TDF Domains to the Intervention Functions most likely to be effective in changing the identified target behaviours, and also provides guidance on identifying salient more fine-grained specific Behaviour Change Techniques (BCTs) to deliver the intervention (48).

We will also adopt the Person-Based Approach to design and build the website to deliver the intervention. This approach can provide in-depth understanding of the needs of patients with AI as the intervention users. It can identify the intervention design features that users view as most important and potential usability problems, thus improving acceptability, user engagement and experience, and effectiveness of the intervention (49). A schematic of the study design guided by the BCW and Person-Based Approach is presented in **Figure 2**.

### 2.2 Phase 1: developing the intervention context (behaviour change wheel)

### 2.2.1 Step 1: define the problem in behavioural terms (BCW Stage 1)

The first step of the BCW involves defining the problem behaviours that need to change and the target population involved in the behaviour, i.e. who is performing the behaviours (45, 46). Published evidence described earlier identified that the problem behaviours performed by patients with AI are related to non-adherence to self-management behaviours in treating their AI on a daily basis, during physical or emotional illness, and preventing and treating adrenal crisis.

#### 2.2.2 Step 2: select the target behaviour (BCW Stage 1)

This step will involve generating a “long list” of all the potential behaviours that may influence the target behavioural problem (45, 46) i.e. self-management behaviours, based on existing evidence.

For patients with AI, the target behavioural problem will involve potential behaviours across the three levels of self-management:

1) Daily management of AI and treatment optimisation to minimise adverse effects from over- or under-replacement;
2) Recognition of “sick days” and appropriate adjustment of glucocorticoid replacement therapy during physical illness and major stressful situations;
3) Adoption of associated behaviours that aid the timely administration of parenteral hydrocortisone to prevent and treat an adrenal crisis.

The “long list” will then be systematically reduced by considering the possible impact of these behaviours on the target behaviour.

#### 2.2.3 Step 3: specify the target behaviour (BCW Stage 1)

Step 3 will involve specifying the behaviour(s) in appropriate detail and in its context in terms of: *who* needs to perform the behaviour, *what* they need to do differently, *when, where, how often* and with *whom* they will do it (46). In this research we will investigate *what* patients with AI (*who*) need to do differently to improve their self-management behaviours on a daily basis, during “sick days” and to prevent and treat an adrenal crisis (*when, where, how often*), and with *whom* they will need to interact to achieve behaviour change.

#### 2.2.4 Step 4: identify what needs to change (BCW Stage 1)

Step 4 will involve a mixed-methods sequential qualitative and quantitative study to identify what needs to change. The aim of this step is to gain in-depth understanding of the barriers and enablers that people with AI experience to performing the target self-management behaviours (qualitative study); this will inform the development and validation of a TDF-based Belief Statements questionnaire to measure the barriers and enablers to self-management in a wider population of patients with AI (quantitative study). The study design is described separately in the following sections for the qualitative and the quantitative studies.

##### 2.2.4.1 The qualitative study (focus group interviews)

###### 2.2.4.1.1 Methods

We will follow the *Consolidated Criteria for Reporting Qualitative Research (COREQ)* checklist (50) to design and report the *Methods* for the qualitative study. Qualitative methods are recommended for behaviour change intervention development when prior evidence is limited (47). This is also in line with the Person-Based Approach which recommends using qualitative research to elicit user views of the planned behaviour changes and exploration of barriers and facilitators (51).

Focus group interviews with patients with AI will be conducted to gain an in-depth understanding of the barriers and enablers to self-management behaviours and to understand what it will take to change the behaviour (achieve the desired behaviour). The benefit of this method is that group interaction encourages participants to explore and clarify individual and shared perspectives, thus eliciting potential elements that can be incorporated into the digital behaviour change intervention.

###### 2.2.4.1.2 Participant selection

Adopting a purposive sampling, we will conduct 7-10 focus group interviews with patients with AI, stratified by AI type (1 pilot group, 2-3 groups for Primary AI, 2-3 groups for Secondary AI, and 2-3 mixed groups including Tertiary AI). Participants will be recruited via the UK-based patient advocacy groups (PAGs), The Addison’s Disease Self-Help Group (https://www.addisonsdisease.org.uk/) and the Pituitary Foundation (https://www.pituitary.org.uk/), using their membership emailing lists, websites, newsletters, and social media; they have a combined international patient membership (primarily UK) of approximately 3,500 members. These PAGs are also collaborators in this study and their representatives are members of the Study Advisory Group.

###### 2.2.4.1.3 Setting

The PAGs will disseminate the study URL link and QR code populated in Qualtrics®, an online secure platform approved for academic surveys and conforming to GDPR regulations, where prospective participants can access the Participant Information Sheet (PIS), eligibility checklist and electronic consent form. Patients who meet the inclusion criteria and provide consent will be allocated to respective focus groups of 6-8 participants per group. The focus groups will be conducted online using a secure university virtual (*Zoom*) account.

Inclusion criteria:

•Adults aged 18 years of age or over
•Diagnosed with irreversible primary, secondary or tertiary AI and on glucocorticoid replacement therapy
•Having internet access to join the *Zoom* call and being comfortable with taking part in online group discussions

Exclusion criteria

•Unable to communicate in English

###### 2.2.4.1.4 Data collection

An interview guide will be developed based on the COM-B model and TDF domains (46, 47), which will be piloted with the first focus group. Open higher-level COM-B questions on Capability, Opportunity and Motivation will act as filters to potentially relevant TDF domains (47). This will be followed by specific questions and probes within TDF domains to collect data that can address the target behaviours in the digital intervention. An example might be:

•“How would you prevent an adrenal crisis?” (COM-B Capability).
•“How do you recognise symptoms and signs of an adrenal crisis for which you would need a hydrocortisone injection?” (prompt in the TDF domain “Knowledge”)

Focus group interviews, lasting 60–90 minutes, will be facilitated by the first author (SL) and another member of the research team and will be video recorded in *Zoom*. The first author will transcribe and de-identify the interviews, adding relevant non-verbal cues and field notes to the transcripts; participants’ names will be replaced with a study identifier, e.g. “3P5” for participant number five in the 3^rd^ focus group.

###### 2.2.4.1.5 Data analysis

A “coding guideline” will be developed in discussion with the research team with explicit statements as to how the TDF is to be applied to the qualitative data (47) using example quotations from the pilot focus group interview; SL and KM will also code the pilot interview jointly to minimise potential discrepancies in the analysis approach. Data will be analysed using NVivo_V12 QDAS qualitative data analysis software following each focus group interview using deductive framework analysis (52) and inductive thematic analysis (53) as below:

•Deductively: Data will be coded using the Theoretical Domains Framework V2 (TDF) as the framework for content analysis (47).
•Inductively: After coding data into theoretical domains, themes and belief statements will be generated to describe similar underlying perspectives and beliefs from respondents which reflect barriers and enablers to target behaviours.

Inductive thematic analysis will also be adopted to generate potential new themes for quotations that may not describe self-management behaviours and do not fit into any of the TDF domains. Data analysis and coding will be performed by the first author (SL); KM who is a COM-B and TDF expert will double-code two focus group interviews. Reliability will be calculated using *kappa* score and assessed by concordance of themes. Findings will be discussed and agreed with a third member of the research team (SH).

The TDF belief statements derived from the focus group interviews will inform the development of a TDF questionnaire which will be used to measure the target self-management behaviours in the Quantitative study. An independent TDF expert will map the generated belief statements to TDF domains to test content validity. The TDF questionnaire will also be reviewed by the Patient Advisory Group comprised of 5 patients with AI and will be finalised in consultation with the Study Advisory Group. Focus group participants will be invited to complete the questionnaire in a pilot online survey and to provide feedback that will inform questionnaire revisions for the Quantitative study. Test-retest reliability will be obtained by administering the TDF questionnaire to focus group participants four weeks later.

##### 2.2.4.2 The quantitative study (cross-sectional survey)

###### 2.2.4.2.1 Study design

We will adopt the *STROBE Statement* (54) and will use the *STROBE Checklist* for cross-sectional studies to design and report the *Methods* in this study protocol. The quantitative study will involve a cross-sectional survey aiming to:

•Measure barriers and enablers to self-management and identify associations with determinants of self-management behaviours in patients with AI.
•Describe the patient education, care and support services available for patients with AI and their families to identify potential gaps and unmet needs.

###### 2.2.4.2.2 Setting and participants

Patients with AI will be invited to complete a cross-sectional survey. The data will be primarily collected online using Qualtrics^®^; however to ensure inclusivity and accessibility the option for postal survey using a prepaid return envelope will also be available. Participants will be recruited via UK PAGs (patient charities) using the same recruitment strategy as for the qualitative study. An eligibility checklist, relying on patients’ self-reporting, will filter participants who meet the inclusion criteria and can proceed with completing the survey. Participants will also be recruited via approximately 15 National Health Service (NHS) endocrine centres invited to the study directly by the research team, via the NIHR Clinical Research Network (CRN) Portfolio, and a call via the *Society for Endocrinology*. Local collaborators (nurse or endocrinologist) in these centres will identify and disseminate the study to potential participants through carrying out a search of patient records based on inclusion criteria.

As the survey will be disseminated via different channels, participants will be advised to complete the survey only once. Cookies will be enabled to prevent participants from completing the survey more than once on the same browser.

Inclusion criteria

•Adult patients aged 18 years or over
•Diagnosed with irreversible Primary, Secondary or Tertiary AI and on glucocorticoid replacement therapy.
•Residing and receiving medical care in the UK.

Exclusion criteria

•Patients with transient AI due to adrenal suppression taking high-dose corticosteroids at the time of study
•Patients not on glucocorticoid replacement therapy

###### 2.2.4.2.3 Variables and *data sources*/*measurements*

The questionnaire, piloted with focus group participants as described earlier, is expected to take 25-30 minutes to complete and will include:

•The TDF Beliefs Statements questionnaire developed from the focus group interviews in the Qualitative study to measure barriers and enablers to self-management behaviours.
•A battery of questions to collect data on clinical and sociodemographic details, patient education, care and support services available for patients with AI.
•The following validated questionnaires which will be used to assess construct validity of the TDF Belief Statements questionnaire and identify potential associations with the three self-management target behaviours:
o The Patient Activation Measure (PAM) (55) to assess the patient’s knowledge, skills, and confidence for self-management
o The Medication Adherence Report Scale (MARS-5) (56) to assess how patients take their glucocorticoid replacement
o The Beliefs about Medicines Questionnaire (BMQ) (57) to assess patients’ beliefs and concerns about their treatment
o The Brief Illness Perception (IPQ) questionnaire (58) to explore how patients perceive their adrenal insufficiency and treatment.

###### 2.2.4.2.4 Bias

Multiple recruitment methods via PAGs and endocrine centres will aim to reduce the response bias often associated with participation from PAG members who are considered more motivated to engage in self-management behaviours compared to the average patient population. Data collection approaches facilitated online and via hard copies can also minimise digital exclusion. In addition, to address recruitment bias and to enable potential generalisability of the findings, we will compare the characteristics of patients responding to the online survey with those of the general population of patients with AI, such as age, sex, AI type, ethnic background, and geographical distribution, which may potentially detect under-representation of some AI patient subgroups. This can further inform the recruitment strategy for the intervention development and usability testing in the next phase of the study.

###### 2.2.4.2.5 Study size

An earlier study recruited 746 patients with AI from PAGs in the UK (59). By extending our study to NHS endocrine centres, we anticipate to recruit approximately 1,500 participants.

###### 2.2.4.2.6 Statistical methods

Quantitative data will be analysed using IBM SPSS Statistics software to determine frequencies, associations and differences between variables and groups, and predictors of self-management behaviours such as adherence to medication and use of preventative measures for adrenal crisis. Qualitative data collected from open-ended questions will be analysed using NVivo using content thematic analysis (53).

#### 2.2.5 Steps 5 and 6: identify intervention functions and policy categories (BCW Stage 2)

Having identified which COM-B components and TDF domains are relevant to the three target self-management behaviours (daily management, “sick days” management, and prevention and treatment of adrenal crisis), we will use the BCW matrix to map the relevant intervention functions such as “education”, “enablement”, “modelling” (*BCW Step 5*) and policy categories such as “guidelines” and “service provision” to consider what policies in the BCW can support the delivery of the selected intervention functions (*BCW Step 6*) based on links between them to select those likely to change behaviour (46).

#### 2.2.6 Steps 7 and 8: identify behaviour change techniques (BCTs) and modes of digital delivery (BCW Stage 3)

In the final two steps of the BCW process, the TDF domains describing determinants of target behaviours identified in the qualitative research and the selected most appropriate intervention functions will be mapped to the Behaviour Change Techniques (BCTs) Taxonomy (v1) (48) matrix to identify a preliminary list of salient BCTs (*BCW Step 7*) and to decide on modes of digital delivery (*BCW Step 8*). The TDF domains and respective barriers and enablers to self-management (TDF Belief Statements) will be prioritised based on (47):

•High frequency of specific beliefs (number of quotes) reported in focus group interviews;
•Perceived importance of these beliefs to self-management as rated by focus group participants on a 1 – 10 importance scale in the pilot study;
•Presence of conflicting beliefs indicating whether these act as barriers or enabler to target behaviours, assessed by the polarisation of Likert scale responses (strongly disagree to strongly agree) to the TDF Beliefs Statement questionnaire in the quantitative study.

The key TDF domains will be mapped to specific BCTs which can be used to address barriers and/or enhance enablers associated with the three target behaviours in a given TDF domain. An Expert Panel of 10-15 key stakeholders (patients with AI, endocrinologists, endocrine nurses and HCI (human computer interaction) practitioners) will be formed to discuss the context of the *Support AI* intervention and to select the most salient BCTs to deliver the intervention. Participants will be sent an information package before the meeting outlining the study objectives and a shortlist of proposed BCTs described in lay language accompanied by example applications (concrete strategy for delivering the BCT) for digital delivery, identified by the research team as the most appropriate to deliver the TDF domains and intervention functions. The content development of the example applications of BCTs will be guided by the supplementary materials accompanying the BCTs Taxonomy (48), by educational materials developed by implementation scientists (60), and by published digital self-management interventions used in other chronic conditions similar to AI such as asthma and type 1 diabetes.

A nominal group technique (61) facilitated by the Chief Investigator (SL) and two experts in TDF and BCTs (KM, SH) will guide the discussions to reach consensus on selected BCTs in steps below:

1. Silent generation: Participants will record their individual response on selected BCTs and their respective example application
2. Participants share ideas in a “round robin” fashion by proposing additional applications for BCTs across the TDF domains for each of the three target behaviours: daily management, “sick days” management, and prevention and management of adrenal crisis
3. Clarification of ideas in an open discussion of feedback bringing in examples and ideas from self-management interventions of other chronic conditions where relevant
4. Participants rank their top 5 BCTs to address each of the three target behaviours.
5. The Panel discusses the top ranked BCTs against the APEASE criteria (Affordability, Practicability, Effectiveness, Acceptability, Safety, Equity) (46).

This will be conducted in two sessions with 2 groups, each with 5-7 participants, over a secure Zoom online account. Points 1-4 will be covered in the first session (2 hours); the research team will analyse findings and the group will reconvene 2-3 days later to discuss point 5 (1 hour). A whiteboard will be used to record participants’ suggestions and ideas which will be used to generate the context of the *Support AI* website. Findings will be discussed with the Study Advisory Group before moving to Phase 2.

### 2.3 Phase 2: designing the *Support AI* digital behaviour change intervention

#### 2.3.1 Aim and methods

•To design an interactive and personalised website underpinned by the Person-Based Approach (49) to address the three target self-management behaviours identified in the earlier phase of this research.

The *Support AI* digital behaviour change intervention (DBCI) will be a responsive mobile-optimised website designed in three iterations described below:

#### 2.3.2 Data collection approach

##### 2.3.2.1 Iteration 1

The Person-Based Approach will inform the creation of guiding principles to describe the key intervention design objectives in terms of behaviour change and the key features of the intervention needed to deliver the behaviour change techniques (49), drawing on findings from the Nominal Group Technique to address the three self-management target behaviours. A list of requirements for the website, i.e. what does the website user need to be able to do, will be generated to help web developers to easily recall and refer to features of the intervention identified in the development phase as central to achieving the intervention objectives.

This iteration will involve deciding the content (what information it should contain) and functionality (what the user should be able to achieve) of the website aiming to produce a low fidelity prototype created using prototyping tools such as Axure® or Figma®. The prototype will include sitemaps (hierarchical structure and navigation of the website pages), wireframes (schematic of the general layout of each page content) and user journeys (a visual representation of the actions the user takes when interacting with the website). Five members of the Expert Panel from the Nominal Group Technique will test the low fidelity prototype moderated by the first author and a UX (user experience) designer.

##### 2.3.2.2 Iteration 2

The web developers supporting the research team will use an “Agile” methodology (42) with 2-week design sprints delivering new modules for review based on the website sitemaps. Adopting a Person-Based Approach and a “think-aloud” technique (49), 12 participants (6 patients, 3 nurses, 3 endocrinologists) will test in real time, using a desktop computer, the high-fidelity prototypes for each module as they are being developed.

This usability testing will evaluate each module for visual design, content and interactivity, iteratively modifying it to optimise user experience. Each “think-aloud” evaluation session will take approx. 60-90 minutes with an estimated total of 12 sessions over 12 weeks (one session per participant). Usability testing will be conducted online and will be video recorded using screen capture.

##### 2.3.2.3 Iteration 3

Following the development of all website modules, 5-7 patients will participate in usability testing of the “alpha version” of the website using “think-aloud” and “screen-capture” techniques as outlined in Iteration 2. Each session, facilitated by the first author and a UX designer, will last approximately an hour and participants will be given specific tasks to complete related to the target behaviours that the *Support AI* intervention aims to address, for example “Find out how to adjust your hydrocortisone tablets if you were ill with the flu”.

Participants will be asked to verbalise what they are doing as they perform each task and to provide feedback on features of the website such as navigation, visual design, functionality, efficiency (62). They will also be asked to complete the Single Use Question (SEQ) 7-point Likert scale (1=very difficult to 7 = very easy) to rate the difficulty of performing each specific task (63). The “think aloud” sessions will be recorded, transcribed and analysed using thematic analysis (53) to identify usability problems.

A “rainbow spreadsheet” and “severity scale” (64) designed in advance, will be used during the observations to record usability problems and feedback on website features (suggestions for improvement, good features, participants’ reactions). Findings will inform revisions of the website for “beta” testing in a future randomised feasibility study.

##### 2.3.2.4 Study sample

Patients with AI will be recruited from the quantitative study of Phase 1; after submitting the survey, they will receive a link to the Participant Information Sheet (PIS) and electronic consent to for Phase 2. Clinicians for Iteration 2 will be recruited via an open call to members of the *Society for Endocrinology*. Naïve participants will be invited for each iteration to minimise bias from prior exposure to the study.

Inclusion criteria for patient participants

•Adults aged 18 years of age or over and receiving medical care in the UK
•Diagnosed with irreversible AI and on glucocorticoid replacement therapy
•Access to the internet and a computer to conduct the usability testing.
•A basic level of digital literacy and competence with navigating a website.

Exclusion criteria

•Unable to read and communicate in English

Inclusion criteria for clinicians

• Involved in providing care to patients with AI in the UK

## 3 Ethical considerations and dissemination

### 3.1 Ethics approvals

Ethics approval was granted by the School of Health Sciences Research Ethics Committee at City, University of London on 6^th^ September 2021 (Reference ETH2021-2215) for the Qualitative Study (focus group interviews) and HRA and HCRW Ethics Approval on 30^th^ March 2022 (IRAS ID: 290622) for the quantitative study and website development.

### 3.2 Participant anonymity and confidentiality

Participants will be provided with a Participant Information Leaflet (PIS) to inform them of the study objectives, expected time commitments, and that participation is voluntary and they can withdraw at any point without an explanation. Survey completion in Phase 1 will denote consent to participate in the study. The online survey will be anonymous with no IP address tracking. Participants will be advised not to use the survey to request medical assistance. Qualitative data will be de-identified by the first author (SL) before analysis to ensure participant confidentiality. There will be no possibility to identify individuals from the published reports, though participants may be able to recognise their responses in the published quotations.

### 3.3 Consideration of participant well-being and remuneration

There are minor anticipated risks associated with this study. Participants will be provided with the contact details of the Chief Investigator and collaborators from each patient advocacy group (PAG) and endocrine clinic should they need to ask questions about the study. They will also be given contact details for the Secretary to the Senate Research Ethics Committee at City, University of London, and local National Health Service (NHS) Patient Advice and Liaison Service for any concerns or potential complaints.

The burden for research participants will be to dedicate approximately two hours of their time to take part in the Qualitative study, 25-30 minutes to complete the survey in the Quantitative study (Phase 1), and up to 90 minutes per session for the website usability testing. Participants in the Nominal Group Technique will need to commit up to 5 hours for participation (2 hours preparation and 3 hours for on-line group discussions). The anticipated risk is that participants may withdraw after providing consent. To mitigate this risk, a detailed description of time commitments and expectations for participation will be provided in the PIS and consent form so participants can make an informed decision. Only naïve participants will be allocated to each stage and iteration to avoid research bias from prior exposure to the study and also to minimise participation fatigue.

Participants will be remunerated for their time as per NIHR and INVOLVE guidance (65), i.e. £20 per hour in the form of high-street vouchers. As the cross-sectional survey is anonymous, it is not possible to provide remuneration to individuals. However, a donation of £3.00 per response will be made to the Pituitary Foundation and the Addison’s Disease Self-Help Group to reward participation.

### 3.4 Data management and storage

Data collected from this study in their original form (digital and hard copies) and in the aggregate pool after screening and removing any identifiable details, will be kept for a minimum of 10 years post study completion. Digital data will be stored in a secure password-protected drive and hard copies will be stored in locked research cabinets at the School of Health and Psychological Sciences at City, University of London. Data collection and storage will conform to the University Policy and will be processed in accordance with the Data Protection Act 2018, GDPR and the Data Protection Bill.

### 3.5 Patient and public involvement and engagement (PPIE)

The PPIE plan for this research conforms to the NIHR INVOLVE National Standards for Public Involvement (66). The Pituitary Foundation and the Addison’s Disease Self-Help Group are collaborators in the proposed study and their representatives are members of the Study Advisory Group who will meet approximately twice a year at Study Milestones. A Patient Advisory Group of 5 patients with AI was also formed to advise the research team throughout the study on the conceptualisation, design, delivery and dissemination of findings.

### 3.6 Dissemination of findings

Findings from the study will be disseminated via scientific meetings, publications in open access peer-reviewed journals and Researchfish® (https://researchfish.com/). The study findings will be disseminated back to participants via the patient conferences, social media and newsletter articles of the participating patient advocacy groups and endocrine centres.

## 4 Study status

As of March 2023, we have completed data collection and analysis from the Qualitative study which involved 51 patients with AI who took part in 10 focus group interviews. Recruitment for the Quantitative study (cross-sectional survey) commenced in December 2022 and will close in October 2023.

## 5 Discussion and anticipated outcomes

Self-management for patients with AI involves complex behaviours across three levels: 1) daily management of the condition and replacement therapy, 2) adjustment of glucocorticoids during “sick days” and 3) adoption of associated behaviours to prevent and treat an adrenal crisis. However, as evidenced by the existing literature, traditional strategies for patient education about these behaviours are not effective. This is the first research study to develop an evidence- and theory-based intervention for patients with AI focusing on behaviour change by targeting the behaviours most relevant to self-management. In line with the MRC guidelines (33) for the design and evaluation of complex interventions such as behaviour change, this research protocol describes the rationale underpinning the development process and maps the intervention components to theory and outcome, providing a fully transparent step by step project design and replicable research methodology.

We have described the systematic process which we will follow using the Behaviour Change Wheel (45, 46) to address determinants of self-management behaviours in patients with AI by qualitatively and quantitively analysing sources of behaviour underpinned by the COM-B/TDF model, linking to the most appropriate intervention functions and policy categories, and subsequently selecting salient behaviour change techniques to use when developing a tailored digital intervention. Adopting a Person-Based Approach (49) and UX (user experience) Design principles with iterative usability testing, we aim to build a digital intervention that is acceptable, engaging, appealing, and easy to use for patients with AI.

In addition, “self-management strategies” is one of the clinical questions being addressed in the National Institute for Clinical Excellence (NICE) guidelines on the management of adrenal insufficiency currently being developed with expected publication in 2024 (67). Given the limited empirical research on self-management in patients with AI, the findings from this study can make a significant contribution to developing evidence-based NICE Guideline recommendations. Future implementation of the *Support AI* intervention in clinical practice can complement usual care to improve patient health outcomes and reduce demand on specialist services.

## Author contributions

SL conceptualised the study, developed the study methodology and drafted the manuscript. SN provides oversight and mentorship for the research activity planning and execution. KM, SH, SW, SB, AG, CN, and SN contributed to the development of the study methodology and provided critical review to the initial manuscript. PS and PM provided PPIE guidance and contributed to the study design. All authors contributed to the article and approved the submitted version.

## References

[1] Bancos I Hahner S Tomlinson J Arlt W . Diagnosis and management of adrenal insufficiency. Lancet Diabetes endocrinology. (2015) 3(3):216–26. doi: 10.1016/S2213-8587(14)70142-1 25098712

[2] Llahana S Mitchelhill I Yeoh P Quinkler M . Diagnosis and management of adrenal insufficiency in children and adults. In: Llahana S Follin C Yedinak C Grossman A , editors. Advanced practice in endocrinology nursing. Cham: Springer International Publishing (2019). p. 705–36.

[3] Broersen LHA Pereira AM Jørgensen JOL Dekkers OM . Adrenal insufficiency in corticosteroids use: systematic review and meta-analysis. J Clin Endocrinol Metab (2015) 100(6):2171–80. doi: 10.1210/jc.2015-1218 25844620

[4] Allolio B . Extensive expertise in endocrinology. adrenal crisis. Eur J Endocrinology. (2015) 172(3):R115–24. doi: 10.1530/EJE-14-0824 25288693

[5] Hahner S Hemmelmann N Quinkler M Beuschlein F Spinnler C Allolio B . Timelines in the management of adrenal crisis - targets, limits and reality. Clin endocrinology. (2015) 82(4):497–502. doi: 10.1111/cen.12609 25200922

[6] Rushworth RL Torpy DJ Falhammar H . Adrenal crisis. New Engl J Med (2019) 381(9):852–61. doi: 10.1056/NEJMra1807486 31461595

[7] Llahana S Zopf K Mitchelhill I Grossman A . Prevention and management of adrenal crisis in children and adults. In: Llahana S Follin C Yedinak C Grossman A , editors. Advanced practice in endocrinology nursing. Cham: Springer International Publishing (2019). p. 1183–205.

[8] Bergthorsdottir R Leonsson-Zachrisson M Oden A Johannsson G . Premature mortality in patients with addison's disease: a population-based study. J Clin Endocrinol Metab (2006) 91(12):4849–53. doi: 10.1210/jc.2006-0076 16968806

[9] Tomlinson JW Holden N Hills RK Wheatley K Clayton RN Bates AS . Association between premature mortality and hypopituitarism. West Midlands prospective hypopituitary study group. Lancet (London England) (2001) 357(9254):425–31. doi: 10.1016/S0140-6736(00)04006-X 11273062

[10] Burman P Mattsson AF Johannsson G Hoybye C Holmer H Dahlqvist P . Deaths among adult patients with hypopituitarism: hypocortisolism during acute stress, and *de novo* malignant brain tumors contribute to an increased mortality. J Clin Endocrinol Metab (2013) 98(4):1466–75. doi: 10.1210/jc.2012-4059 23457412

[11] Hahner S Spinnler C Fassnacht M Burger-Stritt S Lang K Milovanovic D . High incidence of adrenal crisis in educated patients with chronic adrenal insufficiency: a prospective study. J Clin Endocrinol Metab (2015) 100(2):407–16. doi: 10.1210/jc.2014-3191 25419882

[12] Rushworth RL Torpy DJ . A descriptive study of adrenal crises in adults with adrenal insufficiency: increased risk with age and in those with bacterial infections. BMC endocrine Disord (2014) 14:79. doi: 10.1186/1472-6823-14-79 PMC4200115 25273066

[13] HSCIS. Hospital Episode Statistics, Admitted Patient Care - England, 2014-15 . Diagnosis (2015). London: Health & Social Care Information Centre. Available at: http://www.hscic.gov.uk/ (Accessed 10 January 2023). Centre HSCI.

[14] Smans LC van der Valk ES Hermus AR Zelissen PM . Incidence of adrenal crisis in patients with adrenal insufficiency. Clin endocrinology. (2016) 84(1):17–22. doi: 10.1111/cen.12865 26208266

[15] White K Arlt W . Adrenal crisis in treated addison's disease: a predictable but under-managed event. Eur J Endocrinol / Eur Fed Endocrine Societies. (2010) 162(1):115–20. doi: 10.1530/EJE-09-0559 19776201

[16] Chauhan R Lee D . Adrenal insuficiency: burden of disease and cost of illness. value in health (2013). Available at: https://www.valueinhealthjournal.com/article/S1098-3015(13)02556-4/pdf (Accessed 20 November 2022).

[17] Gunnarsson C Ryan MP Marelli C Baker ER Stewart PM Johannsson G . Health care burden in patients with adrenal insufficiency. J Endocrine Society. (2017) 1(5):512–23. doi: 10.1210/js.2016-1064 PMC5686625 29264506

[18] Lovas K Loge JH Husebye ES . Subjective health status in Norwegian patients with addison's disease. Clin endocrinology. (2002) 56(5):581–8. doi: 10.1046/j.1365-2265.2002.01466.x 12030907

[19] Hahner S Loeffler M Fassnacht M Weismann D Koschker AC Quinkler M . Impaired subjective health status in 256 patients with adrenal insufficiency on standard therapy based on cross-sectional analysis. J Clin Endocrinol Metab (2007) 92(10):3912–22. doi: 10.1210/jc.2007-0685 17684047

[20] Bleicken B Hahner S Loeffler M Ventz M Decker O Allolio B . Influence of hydrocortisone dosage scheme on health-related quality of life in patients with adrenal insufficiency. Clin endocrinology. (2010) 72(3):297–304. doi: 10.1111/j.1365-2265.2009.03596.x 19508599

[21] Forss M Batcheller G Skrtic S Johannsson G . Current practice of glucocorticoid replacement therapy and patient-perceived health outcomes in adrenal insufficiency - a worldwide patient survey. BMC endocrine Disord (2012) 12:8. doi: 10.1186/1472-6823-12-8 PMC3403959 22695167

[22] Chapman SC Llahana S Carroll P Horne R . Glucocorticoid therapy for adrenal insufficiency: nonadherence, concerns and dissatisfaction with information. Clin endocrinology. (2016) 84(5):664–71. doi: 10.1111/cen.12991 26641418

[23] Hahner S Loeffler M Bleicken B Drechsler C Milovanovic D Fassnacht M . Epidemiology of adrenal crisis in chronic adrenal insufficiency: the need for new prevention strategies. Eur J Endocrinol (2010) 162(3):597–602. doi: 10.1530/EJE-09-0884 19955259

[24] Filipsson H Monson JP Koltowska-Haggstrom M Mattsson A Johannsson G . The impact of glucocorticoid replacement regimens on metabolic outcome and comorbidity in hypopituitary patients. J Clin Endocrinol Metab (2006) 91(10):3954–61. doi: 10.1210/jc.2006-0524 16895963

[25] Schulz J Frey KR Cooper M Zopf K Ventz M Diederich S . Reduction in daily hydrocortisone dose improves bone health in primary adrenal insufficiency. Eur J Endocrinol (2016) 174(4):531–8. doi: 10.1530/eje-15-1096 26811406

[26] Tiemensma J Andela CD Kaptein AA Romijn JA van der Mast RC Biermasz NR . Psychological morbidity and impaired quality of life in patients with stable treatment for primary adrenal insufficiency: cross-sectional study and review of the literature. Eur J Endocrinol (2014) 171(2):171–82. doi: 10.1530/EJE-14-0023 24801589

[27] Peacey SR Pope RM Naik KS Hardern RD Page MD Belchetz PE . Corticosteroid therapy and intercurrent illness: the need for continuing patient education. Postgraduate Med J (1993) 69(810):282. doi: 10.1136/pgmj.69.810.282 PMC2399661 8321791

[28] Flemming TG Kristensen LO . Quality of self-care in patients on replacement therapy with hydrocortisone. J Internal Med (1999) 246(5):497–501. doi: 10.1046/j.1365-2796.1999.00538.x 10583719

[29] van Eck JP Gobbens RJ Beukers J Geilvoet W van der Lely A-J Neggers SJ . Much to be desired in self-management of patients with adrenal insufficiency. Int J Nurs practice. (2016) 22(1):61–9. doi: 10.1111/ijn.12368 25353148

[30] Burger-Stritt S Kardonski P Pulzer A Meyer G Quinkler M Hahner S . Management of adrenal emergencies in educated patients with adrenal insufficiency-a prospective study. Clin endocrinology. (2018) 89(1):22–9. doi: 10.1111/cen.13608 29617051

[31] Schöfl C Mayr B Maison N Beuschlein F Meyer G Badenhoop K . Daily adjustment of glucocorticoids by patients with adrenal insufficiency. Clin endocrinology. (2019) 91(2):256–62. doi: 10.1111/cen.14004 31050815

[32] Shepherd LM Schmidtke KA Hazlehurst JM Melson E Dretzke J Hawks N . Interventions for the prevention of adrenal crisis in adults with primary adrenal insufficiency: a systematic review. Eur J Endocrinology. (2022) 187(1):S1–S20. doi: 10.1530/EJE-21-1248 35536876 PMC9175553

[33] Craig P Dieppe P Macintyre S Michie S Nazareth I Petticrew M . Developing and evaluating complex interventions: the new medical research council guidance. BMJ (2008) 337:a1655. doi: 10.1136/bmj.a1655 18824488 PMC2769032

[34] Dalgetty R Miller CB Dombrowski SU . Examining the theory-effectiveness hypothesis: a systematic review of systematic reviews. Br J Health Psychol (2019) 24(2):334–56. doi: 10.1111/bjhp.12356 30793445

[35] O'Cathain A Croot L Duncan E Rousseau N Sworn K Turner KM . Guidance on how to develop complex interventions to improve health and healthcare. BMJ Open (2019) 9(8):e029954. doi: 10.1136/bmjopen-2019-029954 PMC6701588 31420394

[36] Bornstein SR Allolio B Arlt W Barthel A Don-Wauchope A Hammer GD . Diagnosis and treatment of primary adrenal insufficiency: an endocrine society clinical practice guideline. J Clin Endocrinol Metab (2016) 101(2):364–89. doi: 10.1210/jc.2015-1710 PMC4880116 26760044

[37] Burger-Stritt S Eff A Quinkler M Kienitz T Stamm B Willenberg HS . Standardised patient education in adrenal insufficiency: a prospective multi-centre evaluation. Eur J Endocrinol (2020) 183(2):119–27. doi: 10.1530/EJE-20-0181 32580144

[38] Harsch IA Schuller A Hahn EG Hensen J . Cortisone replacement therapy in endocrine disorders - quality of self-care. J Eval Clin practice. (2010) 16(3):492–8. doi: 10.1111/j.1365-2753.2009.01149.x 20210825

[39] Repping-Wuts HJWJ Stikkelbroeck NMML Noordzij A Kerstens M Hermus ARMM . A glucocorticoid education group meeting: an effective strategy for improving self-management to prevent adrenal crisis. Eur J Endocrinology. (2013) 169(1):17–22. doi: 10.1530/EJE-12-1094 23636446

[40] van der Meij NTM van Leeuwaarde RS Vervoort SCJM Zelissen PMJ . Self-management support in patients with adrenal insufficiency. Clin endocrinology. (2016) 85(4):652–9. doi: 10.1111/cen.13083 27063934

[41] DoH . The future of healthcare: our vision for digital, data and technology in health and care (2018). London: Department of Health and Social Care Policy Paper. Available at: https://www.gov.uk/government/publications/the-future-of-healthcare-our-vision-for-digital-data-and-technology-in-health-and-care/the-future-of-healthcare-our-vision-for-digital-data-and-technology-in-health-and-care (Accessed 20 February 2023).

[42] West R Michie S . A guide to development and evaluation of digital behaviour change interventions in healthcare. London: Silverback Publishing (2016).

[43] Panagioti M Richardson G Small N Murray E Rogers A Kennedy A . Self-management support interventions to reduce health care utilisation without compromising outcomes: a systematic review and meta-analysis. BMC Health Serv Res (2014) 14:356. doi: 10.1186/1472-6963-14-356 25164529 PMC4177163

[44] NHSEngland . Next steps on the NHS five year forward view (2017). London: NHS England. Available at: https://www.england.nhs.uk/wp-content/uploads/2017/03/NEXT-STEPS-ON-THE-NHS-FIVE-YEAR-FORWARD-VIEW.pdf (Accessed 20 February 2023).

[45] Michie S van Stralen MM West R . The behaviour change wheel: a new method for characterising and designing behaviour change interventions. Implementation Science. (2011) 6(1):42. doi: 10.1186/1748-5908-6-42 21513547 PMC3096582

[46] Michie S Atkins L West R . The behaviour change wheel: a guide to designing interventions. London: Silverback Publishing (2014).

[47] Atkins L Francis J Islam R O’Connor D Patey A Ivers N . A guide to using the theoretical domains framework of behaviour change to investigate implementation problems. Implementation Science. (2017) 12(1):77. doi: 10.1186/s13012-017-0605-9 28637486 PMC5480145

[48] Michie S Richardson M Johnston M Abraham C Francis J Hardeman W . The behavior change technique taxonomy (v1) of 93 hierarchically clustered techniques: building an international consensus for the reporting of behavior change interventions. Ann Behav Med (2013) 46(1):81–95. doi: 10.1007/s12160-013-9486-6 23512568

[49] Yardley L Morrison L Bradbury K Muller I . The person-based approach to intervention development: application to digital health-related behavior change interventions. J Med Internet Res (2015) 17(1):e30. doi: 10.2196/jmir.4055 25639757 PMC4327440

[50] Tong A Sainsbury P Craig J . Consolidated criteria for reporting qualitative research (COREQ): a 32-item checklist for interviews and focus groups. Int J Qual Health Care (2007) 19(6):349–57. doi: 10.1093/intqhc/mzm042 17872937

[51] Yardley L Ainsworth B Arden-Close E Muller I . The person-based approach to enhancing the acceptability and feasibility of interventions. Pilot Feasibility Stud (2015) 1(1):37. doi: 10.1186/s40814-015-0033-z 27965815 PMC5153673

[52] Gale NK Heath G Cameron E Rashid S Redwood S . Using the framework method for the analysis of qualitative data in multi-disciplinary health research. BMC Med Res methodology. (2013) 13(1):117. doi: 10.1186/1471-2288-13-117 PMC3848812 24047204

[53] Braun V Clarke V . Using thematic analysis in psychology. Qual Res Psychol (2006) 3(2):77–101. doi: 10.1191/1478088706qp063oa

[54] von Elm E Altman DG Egger M Pocock SJ Gøtzsche PC Vandenbroucke JP . Strengthening the reporting of observational studies in epidemiology (STROBE) statement: guidelines for reporting observational studies. BMJ (2007) 335(7624):806–8. doi: 10.1136/bmj.39335.541782.AD PMC2034723 17947786

[55] Hibbard JH Mahoney ER Stockard J Tusler M . Development and testing of a short form of the patient activation measure. Health Serv Res (2005) 40(6 Pt 1):1918–30. doi: 10.1111/j.1475-6773.2005.00438.x PMC1361231 16336556

[56] Chan AHY Horne R Hankins M Chisari C . The medication adherence report scale: a measurement tool for eliciting patients’ reports of nonadherence. Br J Clin Pharmacol (2020) 86(7):1281–8. doi: 10.1111/bcp.14193 PMC7319010 31823381

[57] Horne R Weinman J . Patients' beliefs about prescribed medicines and their role in adherence to treatment in chronic physical illness. J psychosomatic Res (1999) 47(6):555–67. doi: 10.1016/S0022-3999(99)00057-4 10661603

[58] Broadbent E Petrie KJ Main J Weinman J . The brief illness perception questionnaire. J Psychosomatic Res (2006) 60(6):631–7. doi: 10.1016/j.jpsychores.2005.10.020 16731240

[59] White KG . A retrospective analysis of adrenal crisis in steroid-dependent patients: causes, frequency and outcomes. BMC endocrine Disord (2019) 19(1):129. doi: 10.1186/s12902-019-0459-z PMC6889201 31791297

[60] Byrne-Davis L Bull E Hart J . Co-Creating behaviour change technique with poeple who will deliver them: the card for change(2022). Available at: https://www.mcrimpsci.org/wp-content/uploads/2018/04/Cards-for-Change.pdf (Accessed 20 February 2023).

[61] McMillan SS King M Tully MP . How to use the nominal group and Delphi techniques. Int J Clin Pharm (2016) 38(3):655–62. doi: 10.1007/s11096-016-0257-x PMC4909789 26846316

[62] Nielsen J . Thinking aloud: the 1 usability tool (2012). Available at: https://www.nngroup.com/articles/thinking-aloud-the-1-usability-tool/ (Accessed 26 February 2023).

[63] Sauro J Dumas J . (2009). Comparison of three on-question, post-task usability questionnaires, in: Proceedings of the Conference on Human Factors in Computing Systems (CHI), Boston, MA.

[64] Sauro J . Rating the severity of usability problems (2013). Available at: https://measuringu.com/rating-severity/ (Accessed 26 February 2023).

[65] NIHR . Payment guidance for researchers and professionals (2022). London: National Health for Health and Care Research. Available at: https://www.nihr.ac.uk/documents/payment-guidance-for-researchers-and-professionals/27392 (Accessed 20 February 2023).

[66] NIHR . UK Standards for public involvement: better public involvement for better health and social care research. 2019 (2019). Available at: https://www.invo.org.uk/wp-content/uploads/2019/11/UK-standards-for-public-involvement-v6.pdf (Accessed 20 February 2023).

[67] NICE . Adrenal insufficiency: acute and long-term management. national institute for health and care ecxellence. guideline in development [GID-NG10237] (2023). Available at: https://www.nice.org.uk/guidance/indevelopment/gid-ng10237 (Accessed 20 February 2023). Expected publication date: 11 April 2024.

